# Experimental and Numerical Studies on Major Pyrolysis Properties of Flame Retardant PVC Cables Composed of Multiple Materials

**DOI:** 10.3390/ma13071712

**Published:** 2020-04-06

**Authors:** Sun-Yeo Mun, Cheol-Hong Hwang

**Affiliations:** Department of Fire and Disaster Prevention, Daejeon University, 62 Daehak-ro, Dong-Gu, Daejeon 34520, Korea; fayamun@gmail.com

**Keywords:** flame retardant cable, pyrolysis reaction, thermo-gravimetric analysis, fire spread, Fire Dynamics Simulator (FDS)

## Abstract

Flame retardant cables were investigated using thermo-gravimetric analysis to measure the reference temperature and reference rate required for a fire spread simulation using a Fire Dynamics Simulator (FDS). Sensitivity analysis was also performed to understand the effects of the reference temperature and rate on the pyrolysis reactions. A two-step pyrolysis reaction was typically observed regardless of the cable type, and each pyrolysis reaction could be attributed to single or multiple components depending on the cable type and reaction order. Although the structures, compositions, and insulation performances of the cables differed considerably, the reference temperatures of the two-step pyrolysis reaction were extremely similar regardless of the cable type. Conversely, the reference rates of the different types of cables varied significantly. The sensitivity analysis results indicate that the mean values of the reference temperature and rate are sufficient to simulate the pyrolysis reactions of flame retardant cables. The results obtained herein also suggest that the heat transfer and pyrolysis reaction path associated with the multi-layered cable structure may be more important for accurately determining the ignition and fire spread characteristics, which are attributable to differences in cable structure, composition, and insulation performance.

## 1. Introduction

Fire spread rate prediction is critical for fire risk assessment and fire safety design, and numerous studies have been conducted on the spread of fire in buildings over the last few decades [1]. In particular, the electrical cables used for various elements in housing and industrial environments pose a constant risk of electrical fires, which account for the largest portion of fires, and the cables themselves can act as ignition sources, inducing large-scale fire or secondary combustibility [2]. As an applied example of electric-cable-induced fire, research on ignition and fire spread due to cable malfunction has been conducted under special conditions such as the microgravity environment of a space station [3,4]. In addition, to prevent fire spread via cable trays installed for power transmission, communication, and measurement in long tunnels, classification and certification tests of cables according to various standards, such as fire spread experiments using cable trays and an experiment with various cable lifetimes, have been conducted [5,6,7].

In order to predict the fire spread rate of a cable stack placed in a cable tray, full-scale fire spread experiments were initially performed on cable trays. In terms of practicality, methods of predicting the combustion characteristics of cables by quantification and empirical formulas for the fire spread rate have been implemented [8,9]. In addition, in hazardous facilities such as nuclear power plants, guidelines on the verification and validation of selected cable models for nuclear power plant applications and fire modeling analysis guidelines have been developed to protect safety facilities and to understand the risk of fire spread in each zone [10,11]. However, the ability to accurately predict fire spread rates based on experimental results under specific conditions is impeded considering the wide variety of fire environments, cable materials and compositions, layouts, and ventilation conditions.

Recently, a method utilizing fire simulations considering a field model was implemented to address these limitations. Fire simulation methods for predicting fire spread rates can be largely divided into simple and pyrolysis models, depending on how the pyrolysis of solid combustibles is considered. The simple model is a basic means of predicting the fire spread rate using the surface ignition temperature of the combustibles and the heat release rate per unit area (HRRPUA). In this model, precise prediction of the surface ignition temperature requires accurate knowledge of thermal properties such as thermal conductivity and specific heat, which can be expressed as functions of temperature [12,13]. In addition, the HRRPUA, which is a required input for fire simulations, is generally measured using a cone calorimeter under a specific heat flux and thus may have a large quantitative difference from the actual heat release rate after ignition. Consequently, the fire spread rates predicted using the simple method differ significantly from actual fire phenomena [14]. Hietaniemi et al. [15] compared the experimental and simulation results for the heat generation and temperature distribution in a space caused by the spread of fire from a simplified cable tray for polyethylene (PE)/cross-linked PE (XLPE) cables. However, although accurate knowledge of the physical properties of materials is required to predict actual fire phenomena, the information available in the literature on multiple and complicated materials has practical limitations.

The pyrolysis model requires detailed information regarding the pyrolysis reaction, such as the activation energy and pre-exponential factors, as well as the thermal properties of the combustibles. Thermo-gravimetric analysis (TGA) is mainly used for this purpose, and pyrolysis reaction information can be obtained directly or indirectly based on the mass change according to the rate at which the temperature rises. Kim et al. [16] investigated the pyrolysis properties by conducting TGA of each component constituting flame retardant cables for trays and performed laboratory-scale fire experiments to obtain property information for cable-induced fire analysis by examining the initial fire characteristics of the cables. Actual cable-induced fires experience multiple reactions with multi-layer and multi-component materials. Consequently, the effects of various parameters that characterize pyrolysis reactions should be investigated. Hostikka et al. [17] applied a genetic algorithm for efficient estimation of numerous parameters and derived factors and reaction pathways related to the pyrolysis reaction of a flame retardant cable. 

Matala et al. [18] investigated a combined method including microscale combustion calorimetry in measuring the heat of combustion of polyvinylchloride (PVC) cables made of polymers and additives as well as TGA results. Although these studies can improve the accuracy of the information about the pyrolysis properties of particular cables, the findings may have practical limitations, given that the materials and compositions of cables differ significantly depending on their purposes. In other words, compiling information about the heat, combustion, and pyrolysis properties of all types of cables for fire spread simulations requires significant time and cost.

The objectives of this study were: to group pyrolysis reactions by TGA for representative flame retardant cables composed of multiple materials, to analyze the characteristics according to the usage and structure, and to investigate the main components for assessment of the pyrolysis reactions of flame retardant cables. The means and standard deviations of the reference temperature and reference rate for the main components of the cables were obtained; further, a practical method of applying pyrolysis properties was suggested for a precise prediction of the fire spread rate regardless of cable type. Finally, one-dimensional (1D) pyrolysis sensitivity analysis performed using the Fire Dynamics Simulator (FDS) verified the applicability of the mean pyrolysis properties of the main components of the cable regardless of cable type.

## 2. Experimental and Numerical Methods

### 2.1. Description of Flame Retardant Cables

**Figure 1:**
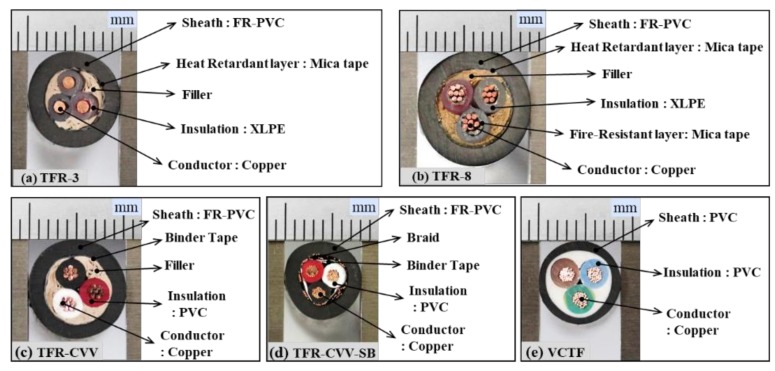
Photographs of cross-section and multiple materials for the cables considered in this study.

Five types of cables consisting of multiple materials were chosen for the pyrolysis property measurements. The cross-sections and constituent materials of each of these cables are shown in Figure 1. The conductors of these cables all have identical specifications (1.5 mm, 2 × 3 C). Figure 1a–d show the cross-sections of the flame retardant control and power cables, which meet the cable burn test standards (IEEE-383) [19]. Specifically, TFR-3 is the tray heat resistant control and signal cable for fire service and TFR-8 is the tray flame retardant power cable for fire service. TFR-CVV and TFR-CVV-SB are tray flame retardant poly vinyl chloride (PVC) sheathed control cables, and TFR-CVV-SB has an additional copper braided shield. Fire protection cables such as TFR-3 and TFR-8 consist of external sheaths of high-performance flame retardant PVC, mica tape known as insulation tape, filler, cross-linked polyethylene (XLPE) insulation, and copper conductors. Control cables such as TFR-CVV and TFR-CVV-SB include external sheaths of high-performance flame retardant PVC, binder tape, PVC insulation, and copper conductors. Figure 1e shows a cross-sectional image of a VCTF cable (PVC insulated flexible cords) consisting of a PVC sheath with insulation provided by a PVC component, which is vulnerable to heat and copper conductors. This cable was examined in comparison with the flame retardant cables mentioned above.

**Figure 2:**
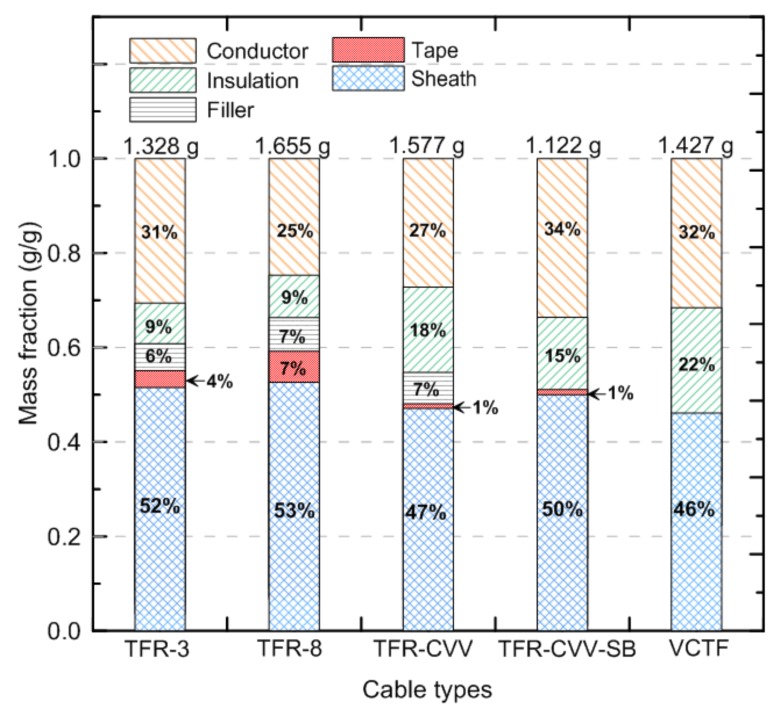
Comparison of the mass fraction of multiple materials, except conductors in the cables.

Figure 2 compares the mass fractions of the constituent materials of the flame retardant cables and VCTF cable, all of which were composed of multiple materials. The mass of each material was measured after cutting a 0.01-m-long section of cable in the longitudinal direction. Although the amount of conductor was the same in all of the cables, the total mass of each cable, excluding the conductor, was different, as shown in the figure. Among the materials that could be classified as combustible, excluding the conductors, the sheath enclosure constituting the surface of the cable occupied the largest portion, with a mass fraction of 65–75%. These results suggest that the heat generated by a cable fire is mainly caused by the sheath, although information about the heat of combustion of each material, which may have multiple components, is not available. The insulation materials have the next-highest mass fraction, ranging from 12% to 33%. The mass fraction of the fillers and tape is relatively low, between 0% and 9%. For reference, the VCTF cable does not include components such as filler or tape.

### 2.2. Experimental Conditions

TGA was performed to characterize the pyrolysis reaction of the cable under changing thermal conditions. TGA is mainly applied to examine pyrolysis characteristics by observing the change in mass of solid combustible materials as a function of temperature. The measurements were performed on a TGA/DSC1 instrument from Mettler-Toledo, and the mass and resolution of the specimen were 5 mg and 1 μg, respectively. The heating rate and maximum heating temperature were 10 °C/min and 650 °C, respectively. Pure pyrolysis without gas-phase ignition was ensured by conducting the measurements in a nitrogen environment. For a heating rate of 10 °C/min and a sample mass of less than 10 mg, the error range of the reaction rate at maximum temperature is known to be less than 5% [20]. The TGA experiments were repeated three times for the same material to ensure reproducibility and to obtain the mean values related to the pyrolysis properties.

The following two-stage approach was considered when investigating the pyrolysis characteristics of each cable. Firstly, the single materials constituting each cable, except the conductors, were subjected to TGA. Secondly, the TGA thermograms of the materials constituting each cable, based on the mass fractions shown in Figure 2, were recorded. Based on these results, it was possible to obtain the temperature at which major pyrolysis occurred and to identify the dominant materials responsible for the pyrolysis.

### 2.3. Evaluation of Pyrolysis Properties and Sensitivity Analysis

A method of applying the complex pyrolysis properties of flame retardant cables obtained experimentally by TGA for fire spread simulation was examined. The 1D pyrolysis model included in FDS version 6.6.0 was used for this purpose. 

The combustion reaction of gaseous fuels generated by the pyrolysis process was not considered, and the 1D conduction heat transfer of solid combustibles was expressed as follows [12]:

(1) $$\rho_{s}c_{s}\frac{\partial T_{s}}{\partial t}=\frac{\partial }{\partial x}\left(k_{s}\frac{\partial T_{s}}{\partial x}\right)+{\dot{q}}_{s,c}^{\prime \prime \prime }+{\dot{q}}_{s,r}^{\prime \prime \prime }\;,$$

where $\rho_{s}$ is the density of the solid combustibles, $c_{s}$ is the specific heat, $k_{s}$ is the thermal conductivity, x is the vertical distance in the depth direction from the specimen surface, $T_{s}$ is the surface temperature on the specimen, ${\dot{q}}_{s,c}^{\prime \prime \prime }$ is the heat release rate per unit volume generated through the pyrolysis reaction, and ${\dot{q}}_{s,r}^{\prime \prime \prime }$ is the absorption and emission of radiant heat per unit volume. 

The boundary condition in Equation (2) was set on the specimen surface (*x* = 0 m), and the adiabatic condition was set on the back of the specimen, as in Equation (3):

(2) $$-k_{s}\frac{\partial T_{s}}{\partial x}\left(0,t\right)={\dot{q}}_{c}^{\prime \prime }+{\dot{q}}_{r}^{\prime \prime }$$

(3) $$-k_{s}\frac{\partial T_{s}}{\partial x}=0,$$

where ${\dot{q}}_{c}^{\prime \prime }$ and ${\dot{q}}_{r}^{\prime \prime }$ indicate the convective and radiative heat fluxes, respectively.

In the pyrolysis model of a solid combustible used in FDS, the reaction rate ($r_{i})$ for the i*^th^* material component can be expressed by the Arrhenius equation as follows [13]:

(4) $$r_{i}=A_{i}Y_{s,i}^{n_{s,i}}\exp\left(-\frac{E_{i}}{RT_{s}}\right),$$

where $Y_{s,i}$ is the ratio of the mass ($m_{s,i}$) to the initial mass ($m_{s}\left(0\right)$) of the combustible, $A_{i}$ is the pre-exponential factor, $n_{s,i}$ is the reaction order with a value of 1 by default, $E_{i}$ is the activation energy, and R is the universal gas constant.

In general, the pyrolysis-related factors for the flame-spread analysis of solid combustibles can be quantified by using TGA. Lyon et al. [21,22] suggested using Equations (5) and (6) to obtain the values of $E_{i}$ and $A_{i}$ required in Equation (4), based on the reference temperature and reference rate, which are two major pyrolysis properties of solid combustibles:

(5) $$E_{i}=\frac{er_{p,i}}{Y_{s,i}\left(0\right)}\frac{RT_{p,i}^{2}}{\dot{T}}$$

(6) $$A_{i}=\frac{er_{p,i}}{Y_{s,i}\left(0\right)}\exp\left(\frac{E_{i}}{RT_{p,i}},\right)$$

where $E_{i}$ and $A_{i}$ are appropriate for multiple-step reactions, the number of peaks in the reaction rate curve is assumed to be equal to the number of material components, and each component is assumed to undergo a single-step reaction that forms a single fuel gas and residue [23]. $T_{p,i}$ and $r_{p,i}/Y_{s,i}\left(0\right)$ are defined as the reference temperature and reference rate (s^−1^) of the i*^th^* material component, respectively. In TGA experiments, these values can be expressed as the temperature at which the peak reaction occurs and the peak reaction rate thereof, respectively. $Y_{s,i}\left(0\right)$ is the mass fraction of material in the original sample undergoing the reaction. For a single component and single combustible material, $Y_{s}\left(0\right)$ = 1. $\dot{T}$ is the heating rate (K/s in Equation (5)) applied in the TGA experiment, and units of K/min were employed in the FDS simulation.

In this study, the dominant reference temperature and reference rate were determined by using TGA to examine flame retardant cables composed of multiple materials. Based on these values, sensitivity analysis was performed to determine the effect of the differences in these physical quantities on the pyrolysis, taking into account the differences among the cables. In the examination of the 1D pyrolysis characteristics using the FDS, a radiative heat flux of 50 kW/m^2^ was applied to the surface of the specimen. The specimen thickness was 0.005 m, and insulation (foam glass) with a thickness of 0.01 m was additionally installed below the specimen.

As shown in Figure 1 and Figure 2, the mass fraction of the sheath with the PVC component was the highest among all of the cables. Hence, the cable material was assumed to be PVC in the sensitivity analysis to determine the reference temperature and reference rate of the pyrolysis reaction. This approach is considered to be realistic because of the limited information on the thermal and combustion properties of cable materials with multiple components. Table 1 lists the properties of the PVC and foam glass required for sensitivity analysis using the FDS. Based on a specimen with a square surface with side lengths of 0.1 m, the initial total mass of the PVC specimen (65.8 g) and foam glass (12.0 g) was 77.8 g.

## 3. Results and Discussion

### 3.1. TGA of the Major Pyrolysis Properties of Flame Retardant Cables

**Figure 3:**
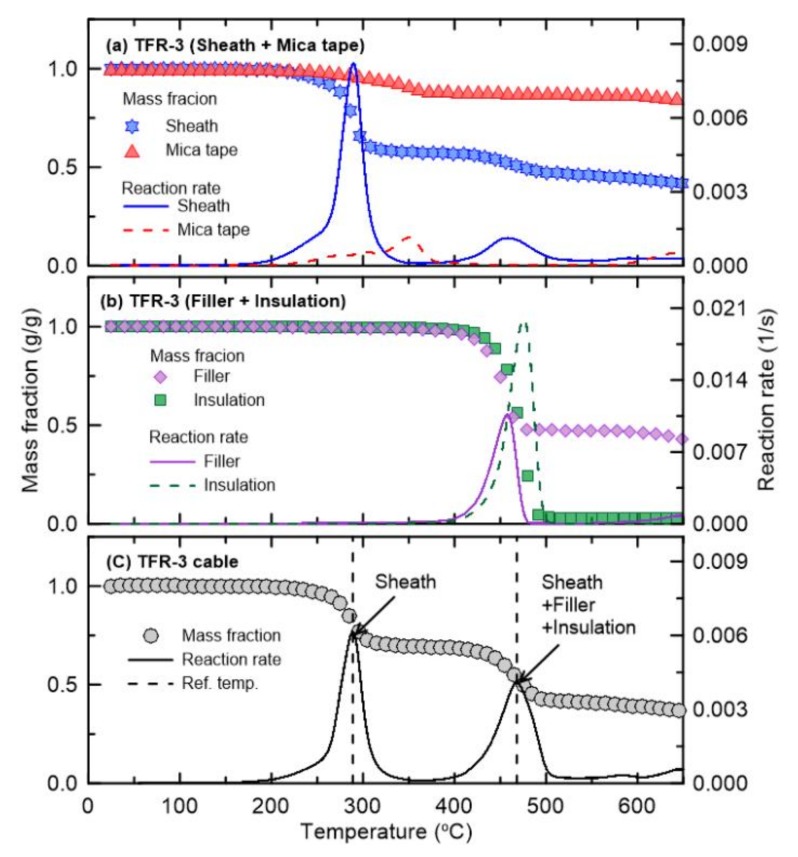
Comparison of mass fraction and reaction rate as a function of temperature for TFR-3 cable.

**Figure 4:**
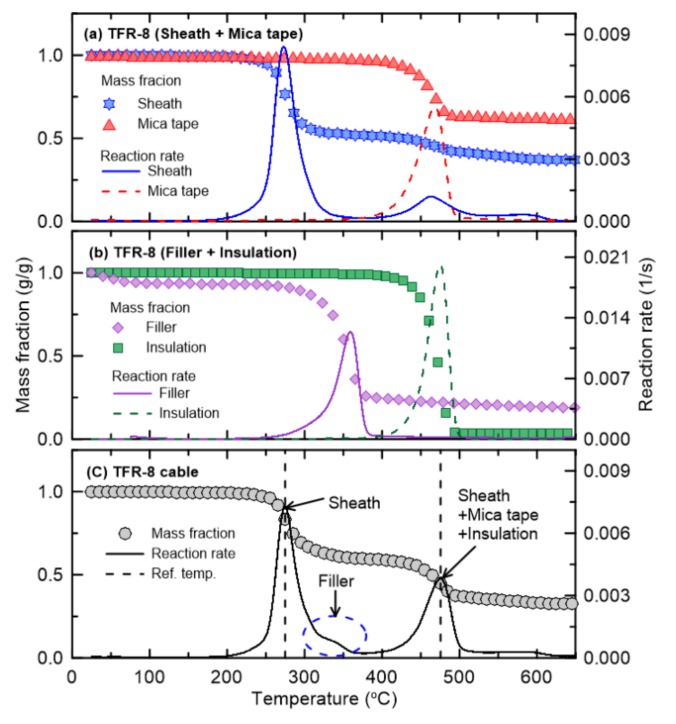
Comparison of mass fraction and reaction rate as a function of temperature for TFR-8 cable.

**Figure 5:**
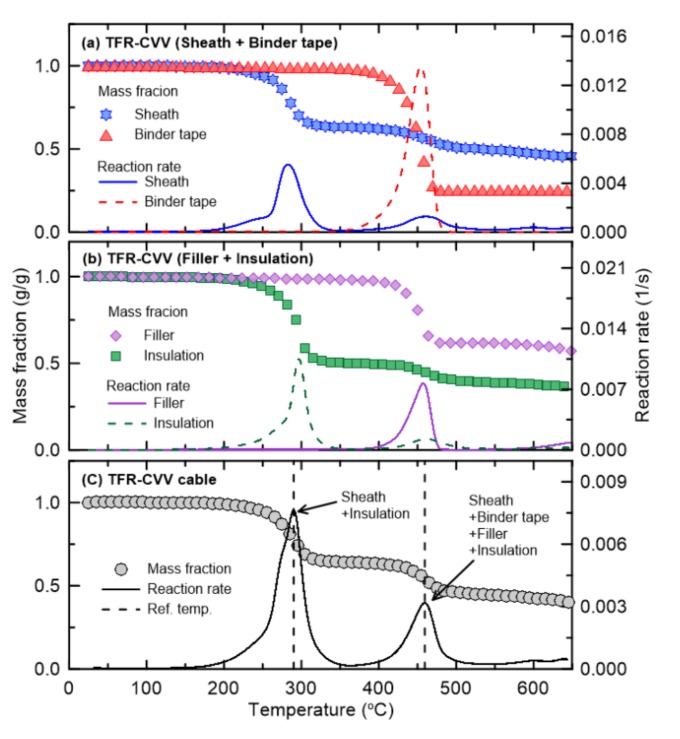
Comparison of mass fraction and reaction rate as a function of temperature for TFR-CVV cable.

**Figure 6:**
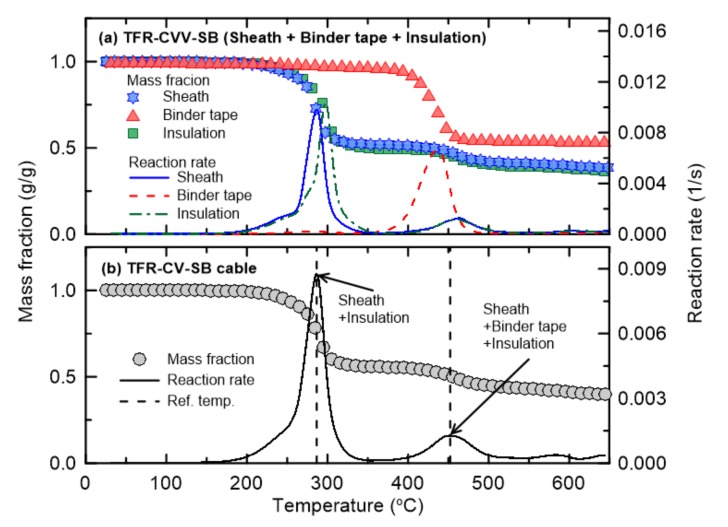
Comparison of mass fraction and reaction rate as a function of temperature for TFR-CVV-SB cable.

**Figure 7:**
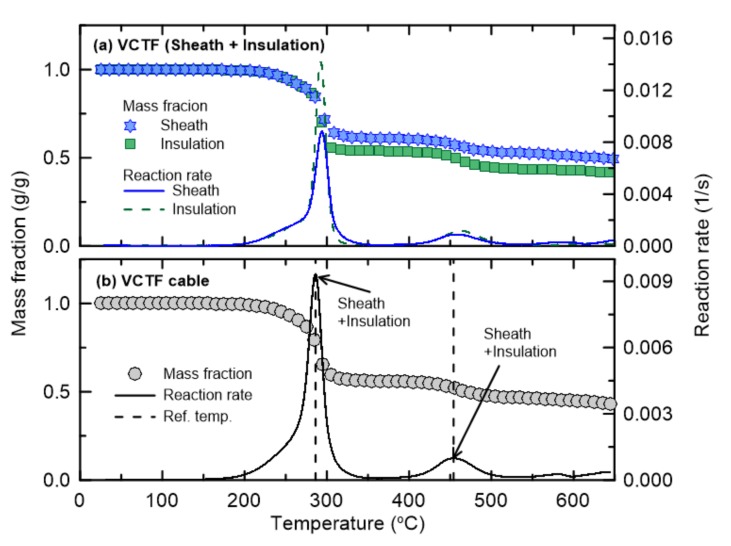
Comparison of mass fraction and reaction rate as a function of temperature for VCTF cable.

The pyrolysis properties required for flame retardant cables composed of multiple materials, i.e., the reference temperature and reference rate, were obtained by TGA. Figure 3, Figure 4, Figure 5, Figure 6 and Figure 7 present the TGA results for the five cables considered in this study as well as the mass fraction and reaction rate as functions of temperature. As mentioned in Section 2.2, TGA was performed in two stages. Firstly, the single materials composing each cable were analyzed separately. Secondly, the multiple materials corresponding to the mass fractions shown in Figure 2 were analyzed simultaneously. Based on this analysis approach, the dominant reference temperatures and rates of the pyrolysis reactions of flame retardant cables composed of multiple materials were determined.

Figure 3 shows the results of the TGA of TFR-3, which was the tray heat resistant control and signal cable for fire service. Figure 3a,b show the mass fractions and reaction rates as functions of temperature for the sheath, mica tape, filler, and insulation. The sheath with the highest mass fraction exhibits a two-stage mass reduction, i.e., a two-step reaction, depending on the temperature change. Although the sheath is known to consist primarily of PVC, it can be interpreted as containing two components, assuming that each component undergoes a single-step pyrolysis reaction [23]. Quantitatively, the reference temperatures at which the pyrolysis reaction of the sheath occurs the most rapidly are 288 °C and 475 °C, and the maximum reaction rates at these temperatures (i.e., the reference rates) are 8.17 × 10^−3^ s^−1^ and 1.12 × 10^−3^ s^−1^, respectively. PVC is well known to produce hydrogen chloride via a pyrolysis reaction in the temperature range 250-300 °C in air or nitrogen [25], corresponding to the first-order reaction in Figure 3a. The reference temperature of the mica tape is 349 °C, which is between the reference temperatures of the two components of the sheath. The reference rate of the mica tape is considerably lower than that of the sheath. For the filler and insulation, the reference temperatures are 457 °C and 475 °C, with reference rates of 1.06 × 10^−2^ s^−1^ and 2.00 × 10^−2^ s^−1^, respectively. These reference temperatures are similar to the temperature related to the second-order reaction of the sheath, and the reaction rates are higher than those of the sheath. It should be noted that XLPE insulation is generally used for fire protection cables, and the initial pyrolysis reaction occurs at a significantly higher temperature than that of the sheath consisting mainly of PVC. Figure 3c shows the results of the simultaneous analysis of the four materials comprising TFR-3 according to their mass fractions. In the figure, TFR-3 exhibits a two-step pyrolysis reaction, with reference temperatures of 284 °C and 464 °C. Comparison of the individual results for each material (Figure 3a,b) clearly indicates that the first-order reaction of TFR-3 is caused by the first-order reaction of the sheath. The second-order reaction takes place over a wider temperature range than the first-order reaction and occurs in the temperature range in which the second-order reaction of the sheath and the reactions of the filler and insulation occur. In other words, the second-order reaction of TFR-3 is presumed to coincide with the effects of the sheath, filler, and insulation. The mica tape does not significantly affect the overall pyrolysis reaction of TFR-3 because of its very low mass fraction. In summary, the pyrolysis reaction of TFR-3 can be expressed as a first-order reaction by a specific component of the sheath and a second-order reaction by multiple materials, including the sheath, filler, and insulation.

Figure 4a,b show the mass fractions and reaction rates with respect to temperature for the individual materials (the sheath, mica tape, filler, and insulation) constituting TFR-8, which is a tray flame retardant power cable for fire service. The changes in the mass fraction and reaction rate with temperature are quite similar to those of TFR-3, except that the reference temperature of the mica tape is similar to the temperature at which the second-order reaction of the sheath takes place. In addition, the reference temperature of the filler is approximately 350 °C, which is lower than that of 457 °C for TFR-3. Figure 4c shows the results of the simultaneous analysis of the four materials comprising TFR-8 according to the mass fraction. As in TFR-3, the first-order reaction corresponds to the pyrolysis reaction exhibited by a specific component of the sheath. However, the second-order reaction is caused by multiple materials, i.e., the sheath, mica tape, and insulation. In addition, the filler in TFR-8 causes a fine inflection point between the temperature at which the first and second reactions occur, unlike the effect of the filler that contributed to the second-order reaction in TFR-3. These results indicate that these two types of fire protection cables (TFR-3 and TFR-8) undergo two-step pyrolysis reactions. That is, the first-order reaction is that of a specific component of the sheath, and the second-order reaction occurs as a result of simultaneous pyrolysis of multiple materials. The second-order reaction is that of multiple materials, including the sheath and insulation, regardless of the cable type. This reaction is also associated with the mica tape and filler, which depend on the cable type. The mass fractions of these two materials are very low, as shown in Figure 2; thus, it seems reasonable to disregard the effects of the tape and filler on the major pyrolysis reactions of the cables.

Figure 5 and Figure 6 show the TGA results of TFR-CVV and TFR-CVV-SB, which are the tray flame retardant control cables with PVC sheaths. Note that because the TFR-CVV-SB cable does not contain filler, it is not considered in Figure 6. As in the fire protection cables, the pyrolysis of the control cables occurs via a two-step reaction. However, the insulation of the control cables undergoes a two-step pyrolysis reaction unlike that of the fire protection cables, because the main component of the insulation surrounding the control cables is PVC, which is similar to that of the sheath. In other words, in the fire protection cables, only the sheath undergoes the first-order reaction, but in the control cables, this reaction is caused by both the sheath and interior insulation material. The second-order reaction of the TFR-CVV cable occurs as a result of the simultaneous pyrolysis of multiple materials (the sheath, binder tape, filler, and insulation). On the other hand, in the case of the TFR-CVV-SB cable, multiple materials (the sheath, binder tape, and insulation) participate in the pyrolysis reaction.

Figure 7 shows the mass fractions and reaction rates as functions of temperature of the sheath and insulation of the VCTF cable. This cable also exhibits a two-step pyrolysis reaction, similar to the fire protection and control cables. Because both of these materials have PVC as their main components, the reference temperatures for the individual materials (Figure 7) and for multiple materials with their corresponding mass fractions (Figure 7b) are quite similar. These results confirm that all of the cables considered in this study undergo two-step pyrolysis reactions. In addition, each pyrolysis reaction can involve single or multiple materials depending on the cable type and reaction order. However, assuming that each material (or specific component) undergoes a single-step reaction [23], the composition of each cable can be simplified as two artificial materials, even when there are multiple components.

**Figure 8:**
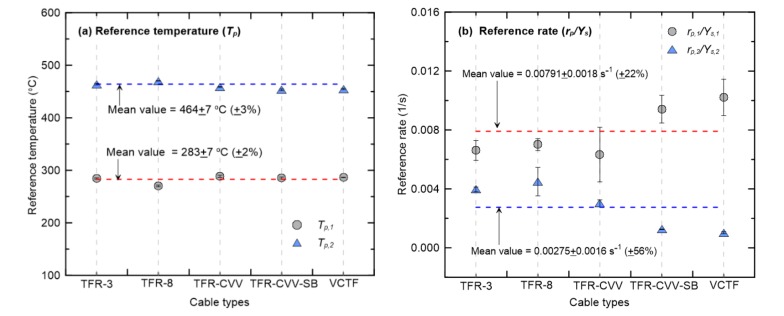
Reference temperatures and rates of the two dominant pyrolysis reactions for flame retardant cables considered.

Table 2 summarizes the TGA results of the five cables considered in this study, showing the reference temperatures and rates in the two-step pyrolysis reaction, which are required as the input parameters for the fire spread simulation. All of the data are presented as the mean values of three repeated experiments. To facilitate understanding of these quantitative values, Figure 8 shows the reference temperatures and rates of the two dominant pyrolysis reactions (R1 and R2) of each cable. The structures, compositions, and insulation performances of the five cables considered in this study differ considerably, except for the conductor specifications. Nevertheless, the reference temperatures of the two-step pyrolysis reaction as presented in Figure 8a are quite similar regardless of the cable type. Quantitatively, the mean reference temperatures for R1 and R2 are 283 °C and 464 °C, respectively, and the standard deviations are identical at ±7 °C. That is, the standard deviations are approximately ±2% and ±3% of the mean values. On the other hand, the reference rates presented in Figure 8b vary considerably depending on the cable type. The standard deviations for R1 and R2 are ±22% and ±56%, respectively, of the mean values. In terms of the reference rate of the first-order reaction caused by the sheath or insulation, TFR-CVV-SB and VCTF have higher values than the other cables. In all of the cables, the sheath and insulation have a common primary component, PVC. However, as shown in Figure 2, the sums of the sheath and insulation mass fractions for TFR-CVV-SB and VCTF are larger than those of the other cables, resulting in higher reference rates. On the other hand, in the second-order reaction, TFR-CVV-SB and VCTF have lower reference rates than the other cables. This difference is evident because the effects of additional materials, except for the sheath and insulation, do not apply to TFR-CVV-SB and VCTF, considering that the second-order reaction is caused by multiple materials almost simultaneously. In summary, it can be concluded that, regardless of the cable composition, the mean values in this study can be used as the reference temperature required to analyze the pyrolysis reactions of flame retardant cables. However, the mean value of the reference rate, which varies significantly according to the cable type, may have to be used with great caution. To this end, sensitivity analysis was performed on the effect of the changes in the reference temperatures and rates on the mass loss of combustibles due to the pyrolysis reaction.

### 3.2. Sensitivity Analysis of Major Pyrolysis Properties using Pyrolysis Simulation

As discussed in Section 3.1, sensitivity analysis was performed to examine the effects of the reference temperature and reference rate on the pyrolysis reaction by quantitative difference according to the cable type. For this purpose, the 1D pyrolysis model included in the FDS was used, and the PVC material with the highest mass fraction among all of the cables was assumed to be combustible. 

**Figure 9:**
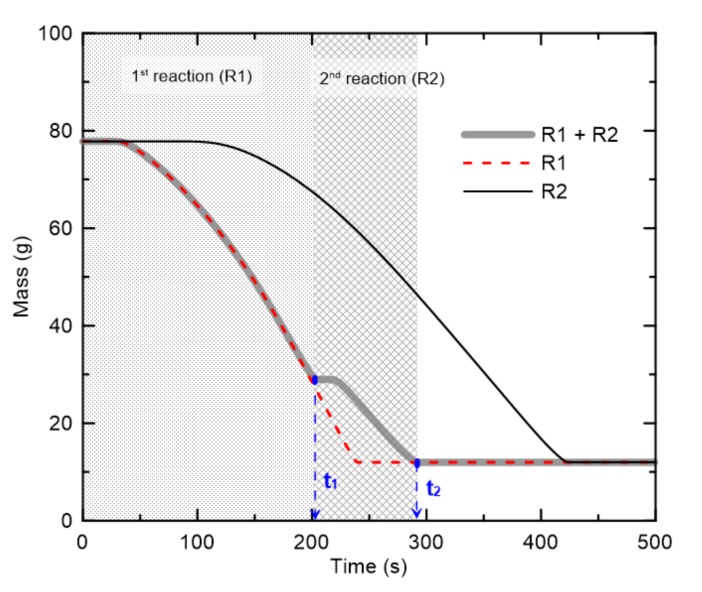
Predicted mass changes when two dominant pyrolysis reactions are considered separately and simultaneously.

Figure 9 shows the change in mass of the PVC material when the reference temperature and rate corresponding to the mean value of all of the cables were applied. To understand the two-step pyrolysis reaction, the mean reference temperatures and rates of R1 and R2 were considered both separately and simultaneously. Based on a square specimen with side lengths of 0.1 m, the initial masses of the PVC and foam glass were 65.8 g and 12.0 g, respectively. Assuming that a material undergoes a single-step reaction [23], when R1 and R2 were considered simultaneously, the initial masses of PVC for R1 and R2 were set to 48.8 g and 17.0 g, respectively. The mass distributions were calculated from the average mass reductions of all of the cables resulting from the two-step reaction. If R1 and R2 are considered separately, it can be seen that the change in mass over time varies significantly depending on the reference temperature and rate of each reaction. When R1 and R2 are considered simultaneously, however, the mass gradually decreases until time *t*_1_ due to R1, and additional pyrolysis subsequently occurs up until *t*_2_ due to R2, which has a higher reference temperature. Note that the mass that remaining after completion of the reaction corresponds to the mass of the foam glass (12.0 g) used as insulation on the rear side of the specimen. The results in Figure 9 show that the effects of the quantitative differences between the reference temperatures and rates of the different cable types on the pyrolysis reaction or mass reduction can be directly assessed by comparing the changes in *t*_1_ and *t*_2_ associated with R1 and R2, respectively.

**Figure 10:**
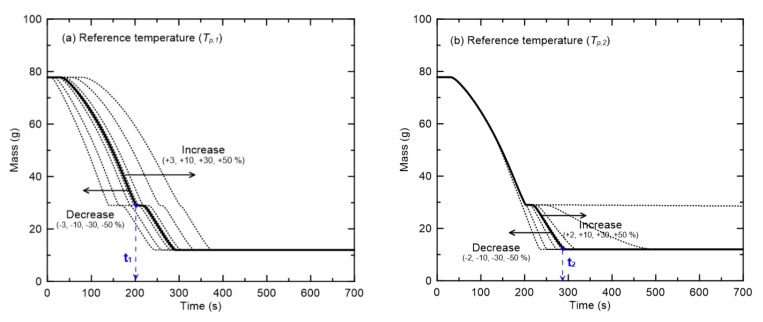
Sensitivity analysis of the reference temperatures in two pyrolysis reactions ((**a**) $T_{p,1}$ and (**b**) $T_{p,2}$) on mass changes.

Figure 10 shows the results of the sensitivity analysis of the effects of changes in the reference temperatures of the two pyrolysis reactions on the mass changes. Here it should be noted that, as shown in Figure 8, the mean reference temperatures (*T_p_*_,1_ and *T_p_*_,2_) of the different cable types were 283 °C and 464 °C for R1 and R2, respectively. Figure 10a represents the mass when *T_p_*_,1_ is changed from ±3% to ±50%, while *r_p_*_,1_/*Y_s_*_,1_ of R1 and *T _p_*_,2_ and *r_p,_*_2_/*Y_s_*_,2_ of R2 remain constant. In this figure, a significant change in *t*_1_ at the end of the first-order reaction due to the change in *T_p_*_,1_ can be clearly identified. In Figure 10b, where *T_p_*_,2_ is changed, a significant change in *t*_2_ corresponding to the end of the second-order reaction is observable when *T_p_*_,2_ is decreased to −50%. Furthermore, when *T_p_*_,2_ is increased to +30%, a large increase in *t*_2_ is observable. In addition, when *T_p_*_,2_ is increased to +50%, the second-order reaction does not occur within 700 s. Thus, it can be concluded that the reference temperature has a considerable influence on the pyrolysis reaction rate. However, as shown in Figure 8a, it should be noted that the standard deviations of the reference temperatures for R1 and R2 are only ±3% and ±2%, respectively. Therefore, the quantitative differences between the reference temperatures of the flame retardant cables examined in this study are expected to have little effect on the pyrolysis rate.

**Figure 11:**
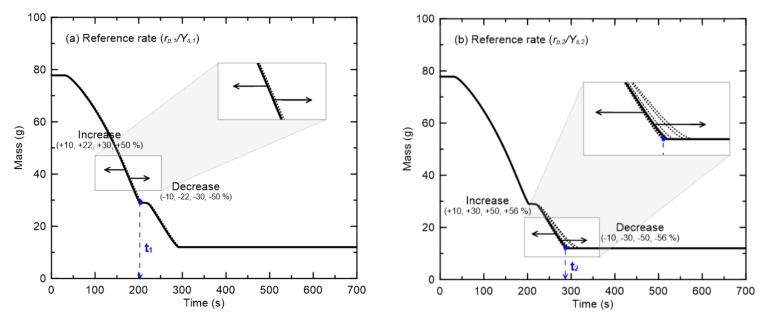
Sensitivity analysis of the reference rates in two pyrolysis reactions ((**a**) $r_{p,1}/Y_{s,1}$ and (**b**) $r_{p,2}/Y_{s,2}$) on mass changes.

Figure 11 presents the results of the sensitivity analysis of the effects of the reference rates of the two pyrolysis reactions (*r_p_*_,1_/*Y_s_*_,1_ and *r_p_*_,2_/*Y_s_*_,2_) on the mass changes. Even if *r_p_*_,1_/*Y_s_*_,1_ for R1 is varied over a wide range, the mass hardly changes because of the first-order pyrolysis reaction. Furthermore, the second-order reaction due to the change of *r_p_*_,2_/*Y_s_*_,2_ also has little effect on the mass. As shown in Figure 8, the reference rates of the flame retardant cables have significantly larger standard deviations than the reference temperatures. However, the large quantitative differences in the reference rates have very small effects on the actual pyrolysis rates.

These results indicate that the mean values of the reference temperature and reference rate obtained via TGA can be applied to simulate the pyrolysis reactions of flame retardant cables. However, they also seem to suggest that the heat transfer and pyrolysis reaction path associated with the multi-layered cable structure may be more important for accurate consideration of the ignition and fire spread characteristics due to the differences in the cable structure, composition, and insulation performance.

## 4. Conclusions

TGA was used to measure the reference temperature and reference rate required to simulate the fire spread behaviors of flame retardant cables. To this end, five flame retardant control and power cables, including one additional VCTF cable (with PVC insulated flexible cords) for comparison, were considered. The differences in the pyrolysis reactions within the standard deviations of the reference temperature and reference rate of each cable were examined by conducting sensitivity analysis using the 1D pyrolysis model included in the Fire Dynamics Simulator (FDS). The major results are as follows:

A typical two-step pyrolysis reaction was observed regardless of the cable type. The components of the cables were subjected to TGA individually and simultaneously. The results showed that each pyrolysis reaction involved single or multiple materials depending on the cable type and reaction order.

Although the fire-retardant cables considered in this study had considerable differences in structure, composition, and insulation performance (excluding the conductor specifications), the reference temperatures of the two-step pyrolysis reactions were similar, regardless of the cable type. Quantitatively, the standard deviations were approximately ±2% and ±3% of the mean values for the first- and second-order reactions, respectively. On the other hand, the reference rates had standard deviations of ±22% and ±56% of the mean values for the first- and second-order reactions, showing significant differences depending on the cable type.

The sensitivity analysis indicated that the reference temperature had a significant effect on the pyrolysis rate, whereas the reference rate had little effect. Considering that the reference temperatures of the cables were quite similar but that the reference rates differed considerably, it can be concluded from a practical point of view that it is sufficient to use the mean values as the reference temperatures and reference rates required to simulate the pyrolysis reactions of flame retardant cables. In addition, the heat transfer and pyrolysis reaction path associated with the multi-layered cable structure may be more important for accurate determination of the ignition and fire spread characteristics attributable to differences in the cable structure, composition, and insulation performance.

## Figures and Tables

**Table 1:** Properties of PVC and foam glass for pyrolysis simulation [24].

Parameter	PVC [24]	Foam Glass [13]
Density (kg/m^3^)	1316	120
Specific heat (kJ/kg·K)	2.0	0.84
Conductivity (W/m·K)	0.25	0.08
Heat of reaction (kJ/kg)	800	-
Heat of combustion (kJ/kg·K)	40,000	-

**Table 2:** Summary of TGA results related to reference temperature and rate for the flame retardant cables.

Cable Type	Reaction Order	Reference Temperature (°C)	Reference Rate (1/s) × 10^−3^
**TFR-3**	R1	284	6.612
	R2	464	3.963
**TFR-8**	R1	270	7.009
	R2	470	4.495
**TFR-CVV**	R1	288	6.326
	R2	459	3.011
**TFR-CVV-SB**	R1	285	9.417
	R2	453	1.252
**VCTF**	R1	287	10.200
	R2	455	1.027
